# Processing of Syndiotactic Polystyrene to Microspheres for Part Manufacturing through Selective Laser Sintering

**DOI:** 10.3390/polym8110383

**Published:** 2016-10-28

**Authors:** Nicolas Mys, An Verberckmoes, Ludwig Cardon

**Affiliations:** 1 Center for Polymer and Material Technologies (CPMT), Faculty of Engineering and Architecture, Ghent University, 9000 Ghent, Belgium; ludwig.cardon@ugent.be; 2 Industrial Catalysis and Adsorption Technology (INCAT), Faculty of Engineering and Architecture, Ghent University, 9000 Ghent, Belgium; an.verberckmoes@ugent.be

**Keywords:** syndiotactic polystyrene, microsphere, rotor milling, spray drying, ball milling, polymer characterization

## Abstract

Syndiotactic polystyrene pellets were processed into powder form using mechanical (ball milling, rotor milling) and physicochemical (spray drying) techniques with the intention of using it as feed material for selective laser sintering. New materials are an important component in broadening the application window for selective laser sintering but must meet strict requirements to be used. Particles obtained were characterized in size and shape using SEM imaging, analyzed by software, and compared to the product obtained by conventional ball milling. Rotor milling and spray drying proved capable of making spherical powders, yet only rotor milling achieved particles with a mean diameter within the desired range of 45–97 µm. Subsequently, the obtained powders were examined for the effect each processing technique imparts on the intrinsic properties of the material. Differential scanning calorimetry analysis revealed amorphization for all methods and a reduction in crystallinity after processing, however, the reduction in crystallinity was acceptably low for the spray-dried and rotor-milled powders. Ball milling displayed an exceptional reduction in crystallinity, suggesting severe degradation. As a final test, the rotor-milled powder was subjected to single-layer test and displayed good coalescence and smooth morphology, albeit with a large amount of warpage.

## 1. Introduction

Selective laser sintering (SLS) is a powder-based additive manufacturing (AM) technique to produce complex 3D parts layer by layer [1]. The development of new powder materials suitable for selective laser sintering constitutes one of the main research topics of today. As the marked share mostly consists of polyamide (PA) materials [2], the application window for SLS remains rather small. Therefore, the introduction of new polymeric materials could broaden this application field. These materials often have to be processed to a powder form suitable for the SLS method, as most production methods do not allow for immediate production of suitable powders for SLS. Goodridge et al. [1] did extensive research on the subject and defined the ideal powder characteristics as dense microspheres in the range of 45–90 µm. Different powder production approaches have already been opted by several authors. Bai et al. [3] reported on the high-energy ball milling of poly(ethylene terephthalate) (PET) both cryogenically and at ambient temperature. Smith et al. [4] did the same but on poly(methyl methacrylate) (PMMA), polyisoprene (PI), and poly(ethylene-*alt*-propylene) (PEP). Both authors commented on the effect of the processing technique on the intrinsic properties of the materials as well as the morphology induced by the processing technique. The ball milling technique often leads to particles with too wide a distribution and undesired morphology [3,5]. Furthermore, these methods often take a lot of time and tend to leave a large amount of waste material that was not fractionated. The use of spheronizers is a widely known method for enhancing the morphology of the pellets, but generally it results in particles with size well beyond the desired range [6]. Other processing methods concern more physicochemical methods like thermal-induced phase separation (TIPS) [7,8,9], diffusion-induced phase separation (DIPS) [8], evaporation phase separation (EPS) [10] and spray drying (SD) [11]. These methods are said to achieve more spherical particles yet require vast amounts of non-solvents to induce the phase separation. Previous work on DIPS and TIPS [12] has already revealed that, in addition to this, particle size was too small (in the range of 1–3 µm) and agglomerations of the particles above a certain weight percentage formed a hurdle that was difficult to overcome. In this regard, the authors of this work propose two novel techniques for the production of microspheres as build material for SLS. 

Spray drying will be the focus of the physicochemical processing techniques, as it virtually eliminates this agglomeration phenomenon by individually drying the atomized droplets. Because of this, one can increase the maximum weight percentage of the polymer solution to be processed, thus increasing the yield. Using an inert loop, the evaporating solvents can be readily recovered, making this an interesting technique as more exotic solvents are often used to dissolve the desired polymers.

An alternative method is suggested for the mechanical processing of the testing material. In this field, rotor milling is tested and compared to the conventional ball milling technique often used in industry. This novel technique is said to be a valuable alternative as it is a short-contact milling method in which the material impacts on a rotary blade at high speed and is subsequently sieved by a built-in sieve of chosen mesh size. The technique minimizes the residence time of the material in the miller, and by doing so, minimizes the possibility of material degradation.

Generally, semi-crystalline materials are preferred over amorphous polymers because of the superior rheology of the powder melt, which causes a better coalescence upon sintering and higher density of the formed object. A common problem of semi-crystalline polymers is their tendency to shrink when they crystallize. In order to overcome this, the polymer in question should exhibit a large processing window [1] to postpone crystallization until after the building process, and thus only show low shrinkage. For this reason, syndiotactic polystyrene (sPS) has been chosen as the testing material. The bulky phenyl groups of sPS prevents close interchain packing of the molecules in the crystal, making the density of the crystalline phase very close to the amorphous phase [13]. Besides this, sPS also displays excellent solvent resistance, making the material useful in any type of application involving acids, bases, oils, water, or steam. The good electrical properties (low dielectric constant (2.6) and low dissipation factor) make sPS suitable for electrical applications such as connectors, plugs, and sockets. Because the material is very light and warpage is less of a problem, sPS can surely substitute for other lightweight metals in their product applications [14,15].

In this study, the authors tested the aforementioned processing techniques (i.e., spray drying, ball milling, and rotor milling) as possible production techniques for microspheres. The powders created in the process were analyzed for particle size and shape as well as any changes occurring in their intrinsic properties due to the processing method. Particle size distributions were established using SEM micrographs, differential scanning calorimetry (DSC) analysis revealed the intrinsic properties of the material, and Hausner ratios were set up by analysis of the powder densities to gain insight to the flowability of the powders.

## 2. Materials and Methods

### 2.1. Materials

sPS XAREC S90Z was provided by Idemitsu Chemicals Europe (Düsseldorf, Germany), unfilled, in the form of pellets and was used as-received. The pellets were dissolved in m-xylene (VWR, Leuven, Belgium, purity 98%).

### 2.2. Solubility Determination

Using the Hansen solubility parameters [16] (HSP), the most suitable solvent was determined to dissolve sPS. The maximum solubility of sPS was determined gravimetrically as the minimum solvent necessary to completely dissolve a weighted sample of sPS, and was expressed in weight percentage (wt %). Dissolution occurred by refluxing sPS at 139 °C (boiling point of m-xylene) until dissolved and left to cool at ambient temperature before further processing. Gravimetrical experiments were replicated three times to minimize experimental errors.

### 2.3. Spray Drying

Spray drying was performed on a Buchi B290 (BUCHI Labortechnik GmbH, Hendrik-Ido-Ambacht, The Netherlands) equipped with a two-fluid nebulizer connected to pressurized air. Nozzle orifice size measured 2.0 mm. The aspirator ran at a maximum air velocity of 40 m^3^/h. An sPS solution of 2.5 wt % was heated to 150 °C and fed to the nozzle using fluoroelastomer tubes at a feed rate of 20 mL/min. The polymer solution was heated to lower the solution viscosity for atomization and avoid crystallization of the sPS in solution [17]. As a measure to further prevent this gelation/crystallization phenomenon, the tubes were preheated by spraying warm solvent prior to spray drying the polymer solution. The heated solution was thereafter atomized using the two-fluid atomizer into the drying chamber using a gas flow rate of 439 L/h. Particles were subsequently dried by a heating gas (pressurized air) which was heated at 200 °C. The resulting powder was sieved off using a cyclone system and investigated.

The critical parameters influencing particle size and morphology were investigated using a systematic approach. Fitting parameters included solution feed rate, nozzle gas flow rate, solution concentration, solution temperature, and heater inlet temperature. Feed rate was controlled by an external peristaltic pump with adapted tubings (Fluoroelastomer tubing, ISO-VERSINIC^®^, VWR, Leuven, Belgium).

### 2.4. Mechanical Milling

#### 2.4.1. Ball Milling

Conventional ball milling was used as a means of comparison to the alternative processing methods described in this paper. Though this technique is typically performed at cryogenic temperatures in order to speed up the communition process, the current setup did not allow for these conditions. Therefore, all milling experiments were executed at ambient temperature. A planetary ball mill (Fritsch Pulverisette type 500, Benelux Scientific BVBA, Eke, Belgium) with three ceramic crucibles and ceramic balls of 25 mm diameter were loaded with 10.1 ± 0.1 g of sPS pellets. Milling was performed at 1400 RPM with samples taken at different intervals. Samples were thereafter examined for size and morphology by SEM (JEOL Europe bv, Zaventem, Belgium) and software analysis by Image J (NIH, Bethesda, MD, USA) and SPSS (IBM, New York, NY, USA).

#### 2.4.2. Rotor Milling

As a second mechanical milling technique, rotor milling was applied. A three-step communition process was set up on a Fritsch Pulverisette 14 (P14, Benelux Scientific BVBA, Eke, Belgium). In the first step the pellets were reduced to a coarse powder of roughly 500 µm by impact milling at this respective size. In the next two steps the powder was subjected to refinement. The second step reduced the powder to 120 µm while the third reduced it further to 80 µm. A 12-ribbed rotor blade was used at 15,000 RPM for this purpose, and in the last communition step this was augmented to 16,000 RPM. The final powder was sieved at 80 µm in order to remove large elongated structures.

### 2.5. DSC Measurements

The thermal properties of the produced powders were investigated using a Netzsch DSC 204F1 (Benelux Scientific BVBA, Eke, Belgium) under nitrogen atmosphere. Samples were contained in an open aluminum pan and referenced against an empty open aluminum pan. A baseline subtraction was done to correct for any slope or variation in heat transfer effects by performing the same measurement with an empty pan both in the reference and sample position and then subtracting the resultant curve. A heating rate of 10 °C/min was used to heat the DSC to 300 °C, and the same rate was used to cool the sample back to RT. The thermal history imparted on the powders by each of the processing methods was investigated using the first heating curve. To explore the reversibility of the structural changes imparted by the processing techniques, a second heating run was explored. Samples were cooled at specific rate in order to induce the α″-form crystallization, which is characterized as a dual melting peak in the thermogram [17]. The crystallinity of each processed sample was determined and compared to the unprocessed sample as a measure to determine degradation. Crystallinity was determined using the following formula:

(1) $$X_{C}=\frac{\Delta H_{f}-\Delta H_{C,\mathrm{cold}}}{\Delta H_{f}^{0}}\times 100$$

where Δ*H*_f_ is the heat of fusion, Δ*H*c, cold is the enthalpy of cold crystallization, and $\Delta H_{f}^{0}$ is the theoretical heat of fusion of sPS displaying 100% crystallinity, which is 53.2 J/g [18].

### 2.6. Particle Size Distribution (PSD)

The morphology of the produced particles was investigated using a scanning electron microscopy (JEOL JSM-7600F, JEOL Europe bv, Zaventem, Belgium) at low voltage (2 kV) and working distance of 8 mm. Samples were sputtered shortly with gold using a sputter coater (SCD 005, BAL-TEC, Wallruf, Germany) at 25 mA. In some cases, optical microscopy (Keyence digital microscope VHX-500F, Keyence, Mechelen, Belgium) was used to determine size and shape of the particles. Obtained micrographs were then analyzed using the software program Image J and further investigated using the statistical program SPSS.

### 2.7. Hausner Ratio (HR)

The Hausner ratio (HR) describes the ratio of the tapped and bulk density of a powder and classifies the powders flowability [19,20]. The HR scale can be subdivided into three main regions [21]:

- HR < 1.25: easily fluidized
- 1.25 < HR < 1.4: decreasing fluidization behavior
- HR > 1.4: fluidization problems

The HR test was performed by gravimetrically measuring the densities of the powder at ambient conditions using a graduated cylinder and powder funnel. After measuring the bulk density, the powder was subjected to a sequence of taps by placing the cylinder on the baseplate of a Retsch Vibratory Sieve Shaker AS 200 digit (Benelux Scientific BVBA, Eke, Belgium) for 60 s at 60% of its maximal amplitude. Afterwards, the volume was measured again to determine the tapped density. To increase its statistical outcome, each powder sample was measured over 10 times (using fresh powder every time), as the HR is highly dependent on the analyst due to the tapping of the powder samples and determination of the volume in the graduated cylinder. The Hausner ratio was calculated as follows:

(2) $$HR=\frac{\rho_{\mathrm{tapped}}}{\rho_{\mathrm{bulk}}}$$

where ρ_bulk_ and ρ_tapped_ are the freely settled bulk density of the powder and the tapped density of the powder in which the powder is tapped until no further changes occur. In this study, the HR of only the rotor-milled samples could be calculated, as the spray-drying and ball milling experiments were conducted on lab scale and did not produce enough quantity to allow HR to be determined.

### 2.8. Single-Layer Testing

As proof of principle the created powders were subjected to a rudimental sinter test. To see if the powders coalesce well upon sintering, single-layer tests were performed in a systematic manner by varying the laser power and scan speed on a 40 watt CO_2_ laser cutter model DC-K401V (Liaocheng Shenhui Laser Company, Shandong, China). Samples were sintered at room temperature, as powder bed heating was not possible. The final sPS powder was spread over a build platform by use of a PMMA knife, after which rectangles of 2 cm × 1 cm were sintered at different scan speeds and laser wattage. The actual temperatures at which the powder was exposed were measured using an IR camera (Testo 875, Ternat, Belgium) with an accuracy of ±2 °C.

## 3. Results

### 3.1. Solubility Determination

Based on the HSP model, m-xylene was chosen as a suitable solvent for sPS. According to Hansen, dissolution should take place when the difference between solubility parameters of solvent (δs) and material (δp) is small (typically δs − δp < 4 MPa^1/2^). This mode of approach has already been discussed in previous paper [12]. A maximum solubility of 4.1 wt % (36.3 mg/mL) was found and verified three times. However, due to the increased risk of gelation at concentrations above 3 wt %, only solutions up to this limit have been used for spray drying. In this study, optimal spray-drying conditions were found at 2.5 wt % solutions of sPS in m-xylene, which will be used for the discussion. Table 1 gives the Hansen solubility parameters of sPS and m-xylene with the maximum solubility achieved in this solvent.

### 3.2. Morphology

**Figure 1:**
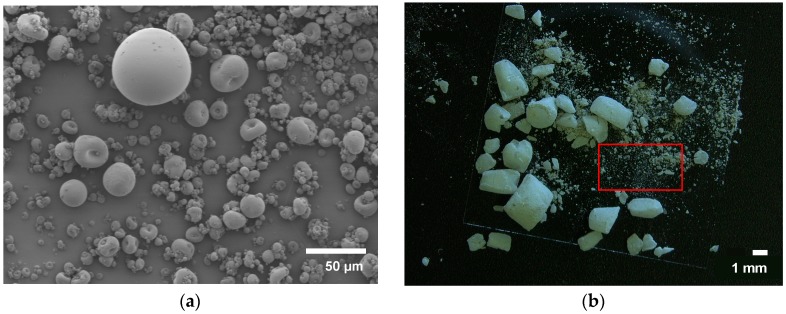
(**a**) Micrograph from spray-dried sPS using the two-fluid nozzle at optimal settings; (**b**) fractionated sPS powder after 15 min of ball milling. The powder marked in the red square was analyzed for PSD (see Section 3.3).

Inspection of the micrographs for the spray-dried sample revealed spherical particles, albeit at too small a diameter (see Figure 1a). A small portion of the microparticles displays a dimpled morphology. This can be explained by the parameter settings, which determine the drying conditions for the particles (see Section 4.1).

**Figure 2:**
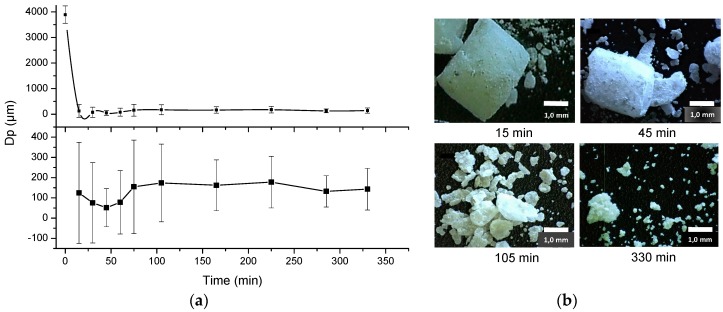
Evolution of average particle size (**a**) and morphology (**b**) of ball milled sPS as function of milling time. The above panel in (**a**) displays the sudden decay in particle size due to immediate fractionation. The lower panel focuses on the fractionation itself. The particle size distribution in the ground material is very broad with angular structures that become flattened into flakes over time. The flattening is clearly visible in (**b**).

When looking at the micrographs and microscopic images for the mechanically milled particles, a big difference is noticeable between the two methods. During ball milling the sample undergoes various morphological changes. In Figure 2, particle size and morphology were plotted versus milling time which depicts this change clearly. After 15 min, already a significant portion of the sPS pellets has fractionated in large rough angular structures and a fraction of very fine particles which were later investigated for PSD (see Figure 1b). All particles are of non-spherical nature. After 45 min, due to the impact of the balls the ground powder begins to flatten into flakes and gets partially cold-welded together upon continuous milling (the low diffusivity of the polymer molecules is expected to hinder this process considerably). This flattening of the particles is noticed in the PSD as well by the small increase in particle size; this is visible in the insets in Figure 2. During the milling process this fractionation of the larger pellets and the flattening of the flakes continues with no apparent improvement of morphology for the use of SLS.

**Figure 3:**
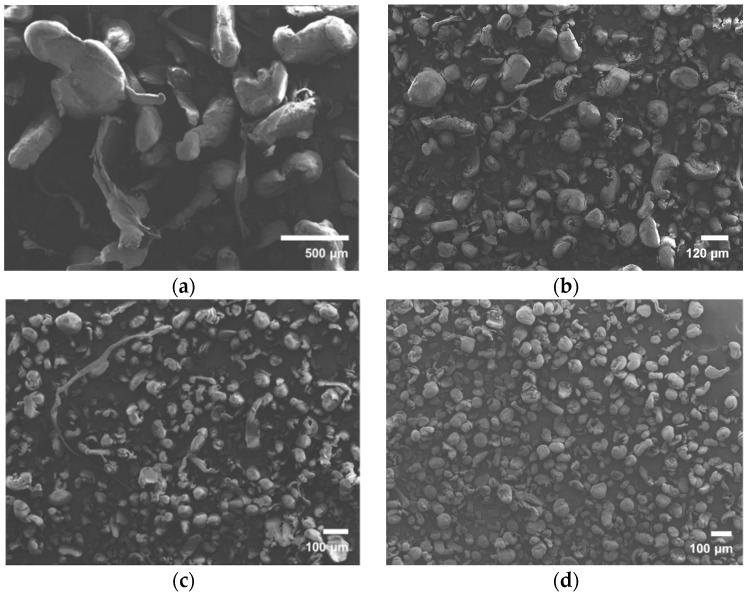
SEM micrographs of the multiple steps of the rotor-milling process. (**a**) Coarse powder by milling sPS pellets at 500 µm; (**b**) first refinement step by milling at 120 µm; (**c**) second refinement step by milling at 80 µm; (**d**) final powder after an additional sieving step at 80 µm.

The rotor-milled powders are prepared in a three-step processing program in which the pellets are processed into a coarse powder and then further refined in two subsequent refinement steps. Micrographs of the powders subjected to each processing step are depicted in Figure 3a–d. A noticeable increase in sphericity is reported with each refinement step. The coarse powder displays little to no spherical structures and is mostly composed of elongated stretched structures (Figure 3a). In the second stage, sphericity is already noticeable, though a large part of the powder remains string-like or as large elongated forms (Figure 3b). Therefore, a third milling step is introduced to further decrease particle size and improve morphology to achieve the desired form (Figure 3c). To remove any unwanted structures, the powder is sieved using a vibratory sieve at 80 µm. The results are fairly spherical particles with smaller inconsistencies (Figure 3d). No indication of cold-welded structures is apparent, as was previously seen in the case of ball milling.

### 3.3. Particle Size Distribution (PSD)

**Figure 4:**
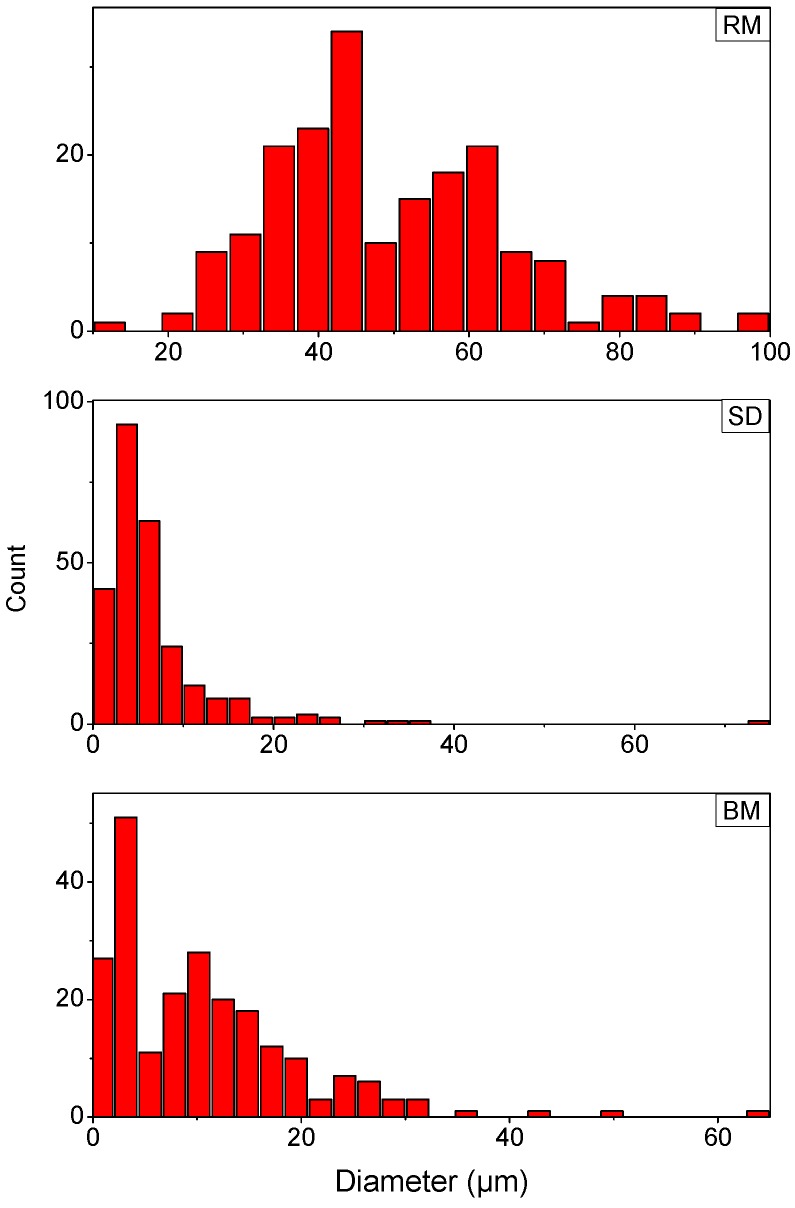
PSD of the differently processed sPS samples. RM depicts the rotor-milled sample (**top**); SD depicts the spray-dried sample (**middle**); and BM the ball milled sample (**bottom**).

Figure 4 displays the particle size distributions of the particles obtained by the three different processing methods. In the case of spray drying, particles were spherical, yet particle size distribution on the micrograph revealed a mean particle size of 6.6 µm with a standard deviation of 6.9 µm and a distribution that is slightly positively skewed. Though some particles reached a diameter within the desired range of 45–90 µm for SLS, the major portion remained well below this. A possible explanation for this could lie in the fact that for these experiments the concentration of polymer remains pretty low. Because sPS is highly crystalline and tends to crystallize from solution by formation of a gel, it is very difficult to spray higher concentrations. Furthermore, the maximum solubility is only 4.1 wt %, which means that only very diluted solutions can be sprayed.

When looking at the graph in Figure 2 for the ball milled sample, an immediate decrease in particle size is visible. Within 15 min the pellets already fractionate, partly resulting in a portion of small and rough angular structures of about 10.6 ± 9.2 µm (see Figure 4) and a portion of bigger, slightly fractionated or yet unfractionated pellets, several orders of magnitude larger. The particle size distribution on the fractionated pellets after 15 min (marked by the red square in Figure 1b) is given in Figure 4. Particle size is already well below the desired range and shows little to no sphericity. After 45 min, a minimum in mean particle size is reached, after which the particle size increases again and levels off. The end result is a fine powder with a PSD ranging around 142 µm with a few particles still as large as 987 µm. During the whole process, particle size distribution is very broad for the as-ground powder but becomes considerably narrower with milling time.

The three-step communition process by the rotor miller decreases particle size sequentially while improving morphology. In the first stage, the pellets are converted to a rough powder with a mean size of 673 µm with a large standard deviation of 245 µm. In the second stage, the particle size is further diminished to a mean size of approximately 73 µm with a standard deviation of 42 µm. In the last processing step, the powders are reduced to a mean particle size of 46 µm with a standard deviation of 21.9 µm. After the final sieving step using the vibratory sieve at 80 µm, a powder is obtained displaying a particle size of 49.2 µm with a standard deviation of 15.6 µm. Figure 4 depicts the PSD of the final product obtained by rotor milling.

### 3.4. Hausner Ratio (HR)

**Figure 5:**
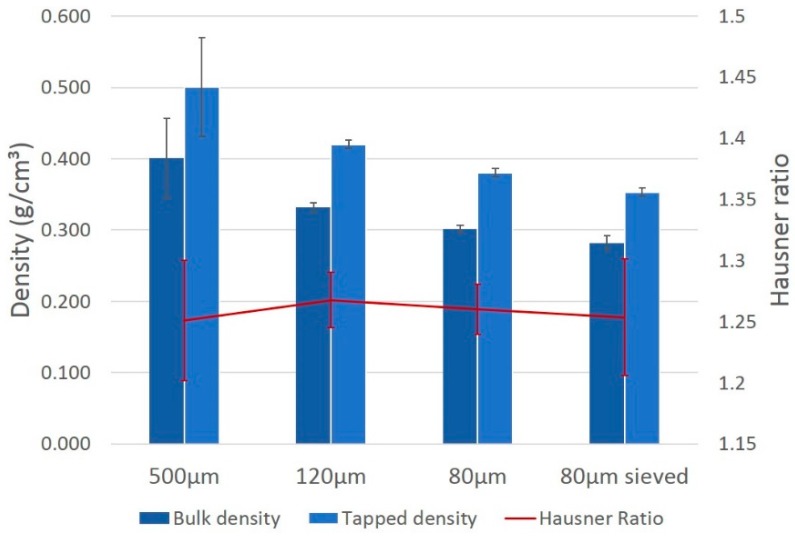
Tapped and bulk density with respective Hausner ratio of each stage in the rotor-milling process.

To investigate the flow properties of the created powder, the bulk and tapped density were determined for each fraction and are represented together with their respective HR in Figure 5. Contrary to what one would expect, the packing density decreases with every step in the rotor-milling process. The HR of the corresponding fractions remain relatively unchanged by the milling steps, and lay in between 1.25 and 1.27 (Table 2).

### 3.5. DSC Measurements

**Figure 6:**
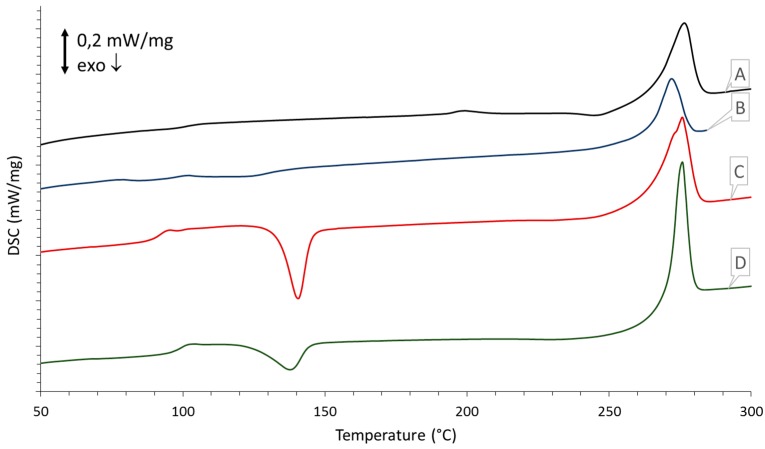
Thermograms representing the first heating run of the processed samples: (**A**) unprocessed; (**B**) ball milled for 15 min; (**C**) spray dried at optimal conditions; (**D**) rotor milled after final sieving step.

**Figure 7:**
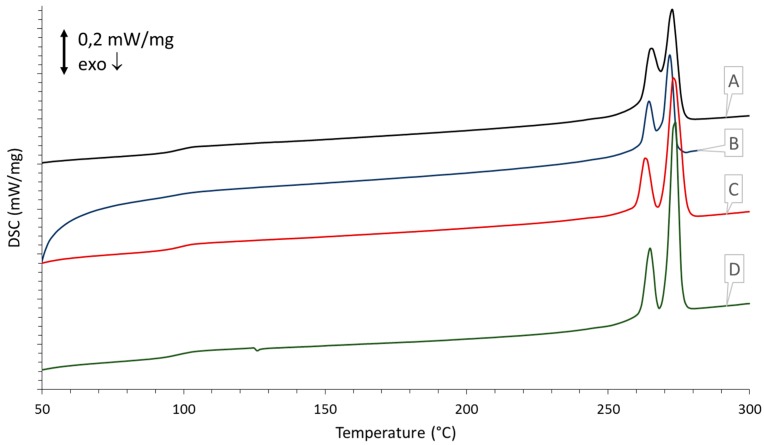
Thermograms representing the second heating runs of the processed samples: (**A**) unprocessed; (**B**) ball milled for 15 min; (**C**) spray dried at optimal conditions; (**D**) rotor milled after final sieving step.

The DSC measurements of the processed samples are given in Figure 6 and Figure 7. In Figure 6 the first heating curves of each processed sample are compared to each other. These curves show the influence each processing technique imparts on the thermal properties of the material and can aid in the setting of the parameters for laser sintering for which these powders are intended. In Figure 7 the second heating curve is depicted, which gives more insight into the intrinsic properties of the polymer and is used to determine whether any degradation has occurred. Table 3 gives an overview of the measured enthalpy values with the resultant percentages of crystallinity calculated according to Equation (1).

Upon processing of the sPS, significant changes in thermal behavior occur. In the case of spray drying, a relatively large cold crystallization peak becomes apparent at 140.4 °C, and the *T*_g_ is found at 92.1 °C but increases back to 100.8 °C in the second thermal run. The broad melting peak at 275.6 °C also deviates a little from the polymorphic profile, as seen with untreated sPS, by the appearance of a small shoulder. Crystallinity of the processed sPS was reduced to 27.3% but increases back to 53.7% in the second heating run. Upon the second thermal run, the thermal history of the processing technique is swept away and a clear α″-structure is recovered, evidenced by the dual melting peaks at 263.8 and 273.3 °C.

When looking at the mechanical processing techniques, a similar trend of amorphization can be seen. In the case of rotor milling, an exothermic peak is detected at 137.7 °C upon heating. The crystallinity of the as-ground powder is lower than its untreated form and shows a crystallinity of 38.5%. A second DSC run reveals a return of the dual melting peaks, though a larger incision is visible between the two characteristic peaks. Crystallinity was calculated at 52.9%, displaying a reduction of 3.4%. Finally, the glass transition temperature was measured at 99.2 °C, in close agreement to the unprocessed sample.

In the case of ball milling a more drastic change in thermal behavior occurs. A more complex exothermic peak is found after the glass transition as a result of amorphization due to the mechanical treatment. The exothermic peak is not as expressed as is the case with the other processing techniques. One might remark that, in the second heating run, the glass transition temperature is slightly lower than usual, measuring 98.1 °C, and could indicate degradation. The crystallinity values of the sample ball milled for 15 min support this premise; a crystallinity of 23.7% and 32.6% is found in the first and second DSC run, respectively, accounting for a significant reduction in crystallinity of 40.5%.

### 3.6. Single-Layer Testing

**Figure 8:**
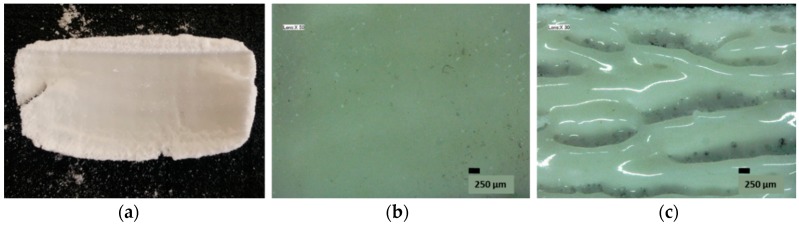
(**a**) Sintered rectangle on 80 µm final powder. Part caking is visible around the rectangle along with extensive warpage. The parts produced at these optimal parameter settings displayed a smooth morphology (**b**) whereas deviation from these parameters often results in nonconsolidated layers or severe necking or balling (**c**) [1,22].

**Figure 9:**
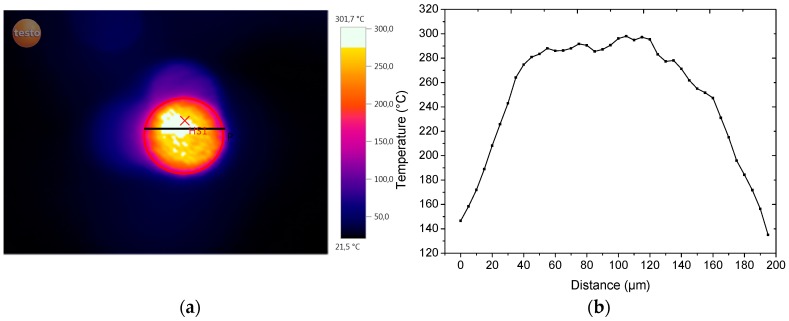
(**a**) Infrared measurements on the laser spot with (**b**) respective temperature profile of the laser spot on 80 µm sieved rotor-milled sPS powder. The temperature profile is obtained by plotting the temperature of each pixel on the black line in (**a**). Temperatures reach well above the melting temperature of sPS.

In an attempt to simulate sinter experiments, a laser cutter was used with variable wattage and scan speed to perform preliminary single-layer tests. A one-factor-at-at-time (OFAT) approach determined the optimal settings at a laser wattage of 20 W and scan speed of 200 mm/s. In this approach, each parameter was systematically changed while the others were kept constant in order to find the optimal settings. In the case of finding the optimal settings of scan speed, the laser wattage was kept constant as the scan speed was varied. The best settings were considered to be those which gave a smooth surface without the visual appearance of laser scan tracks indicative of balling or necking due to full melting particles several layers deep [1,22] (see Figure 8), and this was established to be 200 mm/s. To find the optimal settings for the laser wattage, the scan speed was fixed at 200 mm/s and the wattage was varied. The best settings were considered those in which the powder was sintered completely (no visible unsintered powder at the surface) without any visual form of degradation (fuming). The best wattage setting for the sPS powder sintering in this setup was established to be 20 W. Analysis of the laser spot during sintering with an IR camera revealed temperatures reaching 301.7 °C (Figure 9b), which is well above the melting point of sPS. Severe warpage was observed as well as significant quenching by the production of transparent parts.

## 4. Discussion

### 4.1. Morphology

Powder produced by the spray-drying method consisted of smaller yet more spherical particles compared to the two other methods (see Figure 1a,b and Figure 3d). One would expect all particles to be spherical, as the maximal structural stability of a solution is in its spherical form. However, as polymers tend to have a low diffusion rate due to their large molecular weight, droplets cannot dry out homogeneously in the drying chamber. Instead, a concentration gradient will occur. Due to the low concentration of polymer in solution (only 2.5 wt % was used), further increasing particle diameter without compromising morphology remains difficult. sPS is highly crystalline and tends to crystallize from solution by formation of a gel [17], hampering the spraying of higher concentrations. Furthermore, the maximum solubility is only 4.1 wt %, which means only very diluted solutions can be sprayed. As a result, the creation of bigger droplets by increasing nozzle diameter or feed rate would result in a thinner skin formation and ultimately in more shriveled particles. Vehring et al. [23,24] explains these findings by the use of the Peclet number. Polymers, as a result of their low diffusion rate, exhibit large Peclet numbers, which are often subject to these shriveled morphologies.

Figure 1b and Figure 2 show that a clear change in morphology occurs with longer milling times when ball milling the material. Initially the large part of the pellets fractionates to smaller portions and finer powder, all non-spherical in nature. A minimum is observed at 45 min of milling, after which the diameter increases again and levels off. This increase can be explained when observing the microscopic images on the inset of Figure 2; flattening of the particles occurs, which is accompanied by an increase in particle diameter, as seen after processing with Image J. It is important to note that at the end of the experiment a large portion of partly comminuted pellets still remain and need to be sieved off. From these findings, it is clear that due to the crude processing method, particles fractionate irregularly without the formation of particles within the desired range and morphology. Molina et al. [25] have reported similar results, albeit by grinding amorphous PS with a shaker mill. They reported a final size of 30 µm at which the cold-welding fragmentation of the polymer chips occurs. They confirmed the possibility of a ductile fragmentation phenomenon by local temperature increases caused by deformation under the stress of the beads. 

Rotor milling was chosen as an alternative mechanical milling technique, which reduces residence time of the material in the milling unit and, therefore, reduces chance of degradation. The technique fractionates the material by impacting on the rotor blades, and the particles are further sheared between the rotor and the sieve placed in the milling unit. This extra shearing effect is believed to be the cause of the rounding off of the particles with every sequential milling step. The end result is particles with fairly spherical morphology (Figure 3d). Due to the brittle nature of sPS, not all particles are formed in this way as some tend to fall apart, forming the inconsistencies seen in Figure 3d.

### 4.2. Particle Size Distribution (PSD)

Of the three processing methods, rotor milling, achieves particles with a mean diameter within the desired range (Figure 4). The particle size distribution is slightly positively skewed to the larger diameters. Particles are found to have a mean diameter of 49.2 µm after final sieving with a standard deviation of 15.6 µm. 

As for ball milling, the curve in Figure 2 suggests particles obtain their optimal value for SLS after 45 min, displaying a mean particle size of 51.4 µm with standard deviation of 94.1 µm. However, as one can clearly see from the insets of Figure 2, this average was taken over the entire sample and consisted of the large incomplete fractionated parts and the pulverized powders together. When one investigates the pulverized powder, which already forms after 15 min (Figure 1b), one can see from Figure 4 that this powder consists of particles of 10.6 ± 9.2 µm which are well below the desired range.

Finally, as spray drying proves to be a good technique in terms of optimizing morphology, particle size is severely lacking. Particles with a diameter of 6.6 ± 6.9 µm are unfit for use in SLS processing. Increasing particle size by use of larger more industrial spray dryers [26] could be an option, yet this is hampered by the low solubility of the sPS.

### 4.3. Hausner Ratio (HR)

The Hausner ratio of each milled fraction is displayed in Figure 5. Throughout the milling steps the ratio decreases and ends at the limit of good powder flowability (1.25 for the final sieved fraction [27]). As particle size is decreasing with every milling step, one would expect the density to increase, resulting in a better packing density. However, with every successive milling step the density of the powders decreases. Ziegelmeier et al. [19] noticed a similar behavior when comparing the HR of different thermoplastic polyurethane (TPU) powders and ascribed this to changes in dominating adhesive forces in the powder bulk. The principle reasoning behind this being that, if particle size increases, the weight forces would lower the influence of the van der Waals interactions, allowing the particles to slide more easily alongside each other. These results were enforced by extra powder rheometry measurements regarding avalanche angle measurement and specific energy measurement. As particle size decreases further with every milling step, these van der Waals forces tend to gain influence and could hinder a homogeneous distribution of the smaller particles over the bulk powder. These adhesive forces between the smaller particles can decrease the packing efficiency and lower the density measured.

### 4.4. Thermal Properties

To investigate the influence that the processing methods might impart on the thermal properties of the material, DSC was performed. The first heating run (Figure 6) was used to investigate the thermal behavior of the powders and could be helpful in setting the parameters for laser sintering for which these powders are intended. The second heating run (Figure 7) was used to indirectly detect if any degradation had occurred due to the powderization method used. For this purpose, the degree of crystallization was calculated for the unprocessed and processed forms and correlated to the former (Table 3). As degradation of syndiotactic polystyrene is believed to occur via random chain scission, this initial decrease in molecular weight could impact crystallinity [28,29,30].

All processing techniques impart changes in thermal behavior, which is visible in the first heating run depicted in Figure 6. For spray drying, a cold crystallization peak is visible at 140 °C which is due to large temperature gradient between the drying chamber and the collection vessel in which the microspheres are collected, causing partial quenching to occur. The glass transition is slightly shifted, yet returns to its normal temperature in the second heating run, suggesting there is no significant degradation. Furthermore, the appearance of a shoulder in the polymorphic melting peak suggests partial recrystallization due to the processing technique. The initial decrease in crystallinity can hence be explained by the fast cooling of the particles upon consolidating in the drying chamber and subsequent cooling in the collecting chamber resulting in a more amorphous sPS than the virgin material. The final percentage of crystallinity calculated in the second heating run in Figure 7 expresses a reduction of 1.9% relative to the unprocessed form. This slight decrease once more suggests that there is no significant degradation.

A similar trend of amorphization can be seen in the case of rotor milling. It is well known that mechanical milling can induce amorphization [3]; Ishida et al. [31] studied these effects on Polytetrafluoroethylene (PTFE) and polyethylene (PE) and Castricum et al. [32] confirmed these findings by their research on high-density polyethylene (HDPE). Here, the cold crystallization peak at 137.7 °C can be attributed to amorphization due to the mechanical treatment. Final crystallinity calculated from the second thermal run expresses a deviation of 3.4% from the untreated material. Once more, this could suggest some mechanical degradation though not to a significant extent. Though the milling process only takes up a fraction of seconds, the powders are subjected to multiple treatments to further reduce the particle size, which could have a cumulative effect on degradation behavior. 

In the case of ball milling, a more complex thermal history is found as a result of amorphization due to the mechanical treatment. The exothermic peak is not as expressed as is the case with the other processing techniques. Bai et al. [3] clarified that this could be explained by an oriented morphology induced by the biaxial stresses that are inherent in ball milling. The relaxation of the chains during the heating run could compensate for the exothermic crystallization. In the second heating run the glass transition temperature remains slightly lower than for the untreated sample, suggesting that some degradation occurred during milling. Degradation of the polymer is likely to occur via chain scission produced by the high-energy collisions between polymer and ceramic balls. These chain scissions can create free radicals which may then react with other molecules in the system to produce crosslinks or grafts [28,29,30]. The crystallinity value for the sample calculated in the second heating run supports this premise by expressing a decrease of 40.5% with respect to the virgin polymer. 

Despite the significant exothermic peaks noticed for all processed samples, differences in melting temperatures between treated and untreated sPS remain quite small. All samples have a polymorphic melting peak around 272 °C in the first heating run and clearly regain the dual melting peaks representative of the α″-structure in the second heating run [17]. Crystallinity percentage calculations determined for these α″-structure melting peaks (see Table 3) indicate that none of the processed samples could fully regain the crystallinity of the unprocessed sPS, however, the spray-dried and rotor-milled powders have a crystallinity close to that of the unprocessed sPS. Ball milling exhibited a significant deviation. This sample is considered to be subject to the most degradation.

### 4.5. Single-Layer Testing

Single-layer tests were performed in order to test the sinter behavior of the material and get a preliminary idea of possible sinter parameters. Parts were performed on a laser cutter in open atmosphere at room temperature. As a result of the temperature gradient between laser spot and surrounding atmosphere, the parts experienced severe quenching giving amorphous parts that were transparent. Furthermore, the thermal gradient also caused severe shrinkage and curling of the part. No fuming or discoloration occurred during the sinter process and parts had a smooth surface at optimized parameter settings. Consequently, these preliminary tests were favorable towards further sinter experiments. Powder bed heating is advised in order to prevent amorphization and shrinkage due to crystallization and thermal gradient. 

## 5. Conclusions

Syndiotactic polystyrene pellets were processed to powder form for SLS production purposes both in a physicochemical and a mechanical way. In the case of conventional ball milling, pellets fractionated partly into very fine powders of angular morphology only after 15 min. Particle size investigations revealed a mean particle size of 10.6 µm with a standard deviation of 9.2 µm, which is too small for laser sintering purposes. Furthermore, a discoloration was noticed during sampling which became more prominent with milling time, suggesting degradation. Thermal investigations revealed a splitting of *T*_g_ and a strong decrease in crystallinity when recrystallizing from the melt (up to a decrease of 40.5%). These findings strongly implicate occurrence of degradation during ball milling in a detrimental way, making this method undesirable as a processing method for SLS powders. Rotor milling, however, displayed much better results regarding morphology and particle size. Here, a three-step refinement procedure using sieves of, respectively, 500, 120, and 80 μm was implemented in order to obtain powders with the desired mean particle diameter. An additional sieving step following the milling was effected in order to remove any inconsistencies. Particles with a mean diameter of 49.2 µm with standard deviation of 15.6 µm were reported. Particles were of spherical morphology, though some inconsistencies remained even after the final sieving step. Nevertheless, the Hausner Ratio calculated for the final powder fell within the range of good flowability, which is essential for SLS purposes. Thermal investigation of the sample displayed amorphization due to the mechanical milling method, yet a good recovery of crystallization was reported after crystallization from melt. A decrease in crystallinity of 3.4% was stated as a result of the processing method. Spray drying could offer a good alternative as a physicochemical processing technique, yet it requires more optimization in regards to particle size. Spherical particles were obtained with a mean diameter of 6.6 µm and with standard deviation of 6.9 µm. Due to the fast cooling rate that the droplets undergo when consolidating from solution to microspheres during spray drying, amorphization is a side effect of this technique. This was reflected in the DSC measurements. A reduction in crystallinity of 1.9% was calculated in comparison with the untreated sample. As a final test, the rotor-milled powders were subjected to a rudimentary single-layer test in order to see if good coalescence could occur without severe fuming or degradation. Smooth surfaces could be obtained using a scan speed of 200 mm/s and laser wattage of 20 W, yet warpage of the layers was unavoidable due to the large temperature gradient between laser spot and surrounding atmosphere. Deviation from these parameters often resulted in rough structures not suitable for multilayer testing. 

## Figures and Tables

**Table 1:** Solubility of syndiotactic polystyrene (sPS) and Hansen solubility parameters (HSP) [16].

Product	Solubility (mg/mL)	Hansen’s total δ (MPa^1/2^)	δ_d_ (MPa^1/2^)	δ_p_ (MPa^1/2^)	δ_h_ (MPa^1/2^)
sPS	-	19.26	18.50	4.50	2.90
m-xylene	36.3 ± 0.9	18.01	17.80	0.82	2.66

**Table 2:** Hausner ratios calculated from the density measurements of each rotor milling step.

Fraction	500 µm	120 µm	80 µm	80 µm sieved
Hausner ratio	1.25 ± 0.05	1.27 ± 0.02	1.26 ± 0.02	1.25 ± 0.05

**Table 3:** Enthalpy values calculated from the thermograms in Figure 6 and Figure 7 with the resultant percentages of crystallinity calculated according to Equation (1).

Measurement	Unprocessed	Spray dried	Rotor milled	Ball milled
First heating run				
Δ*H*_c_, cold (J/g)	n.a.	14.90	8.69	1.96
Δ*H*_f_ (J/g)	25.17	29.44	29.16	14.57
% crystallinity (%)	47.30	27.30	38.50	23.70
Second heating run				
Δ*H*_c_, cold (J/g)	n.a.	n.a.	n.a.	n.a.
Δ*H*_f_ (J/g)	29.12	28.57	28.14	17.33
% crystallinity (%)	54.70	53.70	52.90	32.60

## Abbreviations

The following abbreviations are used in this manuscript:

AM	Additive manufacturing
DIPS	Diffusion induced phase separation
DMF	*N*,*N*-Dimethylformamide
DSC	Differential scanning calorimetry
EPS	Evaporation induced phase separation
HDT	Heat deflection temperature
HSP	Hansen solubility parameter
HR	Hausner ratio
IR	Infrared
PET	Polyethylene terephthalate
PI	Polyisoprene
PMMA	Poly(methyl methacrylate)
PSD	Particle size distribution
RPM	Rounds per minute
RT	Room temperature
SD	Spray drying
SEM	Scanning electron microscopy
SLS	Selective laser sintering
sPS	Syndiotactic polystyrene
TIPS	Thermal induced phase separation
